# Drug-Induced Osteoporosis

**DOI:** 10.3390/jcm15030993

**Published:** 2026-01-26

**Authors:** Rudolf Wolfgang Gasser, Roland Kocijan, Afrodite Zendeli, Heinrich Resch

**Affiliations:** 1 Department of Internal Medicine I, Medical University of Innsbruck, 6020 Innsbruck, Austria; rudolf.gasser@i-med.ac.at; 2 1st Medical Department, Ludwig Boltzmann Institute of Osteology at the Hanusch Hospital of OEGK and AUVA Trauma Centre Meidling, Hanusch Hospital, 1140 Vienna, Austria; 3 Metabolic Bone Diseases Unit, Medical Faculty, Sigmund Freud University Vienna, Sigmund Freud Platz 3, 1020 Vienna, Austria; afrodite.zendeli@med.sfu.ac.at; 4 Metabolic Bone Diseases Unit, Head Osteology, Medical Faculty, Sigmund Freud University Vienna, Sigmund Freud Platz 3, 1020 Vienna, Austria

**Keywords:** drug-induced osteoporosis, glucocorticoids, aromatase inhibitors, androgen-deprivation therapy, thyroxine, proton pump inhibitors, anticoagulants (heparin, vitamin K antagonists), antidepressants, neuroleptics, thiazolidinediones

## Abstract

The administration of various medications can induce bone loss as an adverse effect and may result in drug-induced osteoporosis, an important and clinically relevant form of secondary osteoporosis associated with an increased fracture risk. This review summarizes the skeletal effects of selected commonly used drugs with respect to bone metabolism, bone mineral density, and fracture outcomes. Medications may exert direct effects on osteoblasts and/or osteoclasts, leading to impaired bone remodeling and reduced bone mass. Alternatively, indirect mechanisms may contribute to skeletal damage, including disturbances in calcium and vitamin D metabolism with subsequent secondary hyperparathyroidism, as well as therapy-induced hypogonadism. Drug classes frequently associated with drug-induced osteoporosis during long-term use include glucocorticoids, aromatase inhibitors, androgen-deprivation therapy, thyroxine, proton pump inhibitors, anticoagulants (heparin and vitamin K antagonists), antidepressants, neuroleptics, and thiazolidinediones. Importantly, this overview represents a selection of relevant agents and does not aim to provide an exhaustive list. When prescribing potentially bone-damaging medications over extended periods, particularly in older individuals, bone health should be proactively considered. Evaluation should include laboratory assessment, fracture risk estimation (e.g., FRAX^®^), and bone mineral density measurement when appropriate. Adequate calcium and vitamin D intake should be ensured, and guideline-based osteoporosis therapy initiated when indicated.

## 1. Introduction

Osteoporosis is a serious bone disease characterized by reduced bone mass and deterioration of the bone microarchitecture, leading to an increased risk of fractures. A complex interplay of osteoblasts, osteoclasts, and osteocytes leads to a regulated process of bone formation and resorption, whereby in osteoporosis, bone formation cannot sufficiently replace bone resorption. Postmenopausal osteoporosis in women and age-related osteoporosis in women and men is referred to as primary osteoporosis. Secondary osteoporosis occurs as a consequence of clearly defined diseases or when various medications affect bone [1,2]. Bone loss due to pharmacotherapy is considered a major cause of iatrogenic bone damage. As drug-induced osteoporosis, this is an important cause of secondary osteoporosis [1,2,3,4].

Some medications have been shown to directly affect osteoclast and osteoblast activity, resulting in impaired bone remodeling and regeneration. While glucocorticoids (GCs) are assumed to have a bimodal mode of action (increased resorption followed by reduced osteoanabolic activity), other drugs mainly induce increased bone resorption. Some drugs promote the occurrence of osteoporosis or fractures via indirect effects: disturbances in calcium and vitamin D metabolism with the development of secondary hyperparathyroidism leading to bone loss. Treatment-induced hypogonadism is another mechanism that causes osteoporosis. Finally, certain medications can lead to an increased tendency to fall and thus to a higher risk of fractures.

The purpose of this review is to provide a current overview of the impact of important and commonly used medications on bone metabolism, bone mineral density (BMD), and fracture risk, and the derivation of consequences for clinical practice. However, the selection is not complete, as other medications can also be harmful to the bone. Drugs with potentially negative effects on bone, which are discussed in the following overview, are glucocorticoids (GCs), aromatase inhibitors (AIs), androgen deprivation therapy (ADT), thyroxine, proton pump inhibitors (PPIs), anticoagulants (heparin and vitamin K antagonists (VKAs)), antidepressants, neuroleptics (antipsychotics), and antidiabetics (thiazolidinediones (TZDs)).

## 2. Glucocorticoids

Systemic GC therapy represents one of the strongest risk factors for low-traumatic fractures. GC-induced osteoporosis (GIOP) is therefore the most important form of secondary osteoporosis, occurring largely independently of age, sex, or ethnicity. The approval of modern, often antibody-based therapies in inflammatory disorders such as rheumatologic or inflammatory bowel diseases has enabled substantial reductions in GC use in recent years. Nevertheless, it is estimated that approximately 15 million individuals in the European Union alone are treated with GCs on a regular basis [5].

GC therapy exerts detrimental effects on all three bone cell lineages. Osteoblast differentiation is suppressed, mediated in part by increased sclerostin expression and reduced bone morphogenetic protein (BMP)- and WNT-signaling activity. The regulatory osteocytes undergo apoptosis and enhanced Receptor Activator of Nuclear Factor κB Ligand (RANKL) activity promotes osteoclastogenesis and thus bone resorption. In addition, GC therapy suppresses the sex hormones testosterone and estrogen and reduces intestinal calcium absorption [6,7].

The fracture risk under GC therapy is dose-dependent and predominantly affects the vertebral skeleton. Even at a daily GC dose of 2.5 mg prednisone equivalent, an increased risk of vertebral fractures can be observed, with a steep rise in the fracture risk at higher doses. The risk of peripheral fractures is also elevated. Both the occurrence of vertebral fractures and systemic bone mineral density loss may already be detected within 3 months after initiation of GC therapy [8]. Although the BMD and fracture risk may improve after withdrawal of GCs, GC-induced bone loss is often only partially reversible and may not fully recover within one year in many patients, depending on age, menopause, dose, duration, the presence of vertebral fractures, and underlying disease [9,10,11]. Early assessment of the fracture risk and initiation of osteoporosis-specific therapy is therefore mandatory in patients on GCs.

According to the European Calcified Tissue Society (ECTS) recommendations on GC-induced osteoporosis, patients are stratified into the medium-, high-, and very-high-fracture-risk categories based on the fracture history and severity, with recent major osteoporotic fractures—particularly hip, pelvic, or moderate-to-severe vertebral fractures—defining the highest-risk group and guiding the first-line treatment choice (see Table 1). The following measures are advised [8]:

- General recommendation for all GC users > 3 months: normalize calcium and protein intake, ensure sufficient vitamin D status, and reduce fall risk.
- Initial assessment of fracture risk is recommended for all adults ≥ 50 years or patients at high fracture risk prior to or early during GC therapy. This includes vertebral imaging and a GC dose-adjusted fracture risk assessment (FRAX^®^) calculation.
- Pharmacological therapy is indicated in adults ≥ 50 years with (i) fragility fracture, (ii) T-scores ≤ −1.5, (iii) GC dose ≥ 7.5 mg/day, (iv) age ≥ 70 years, or (v) country-specific FRAX^®^ thresholds exceeded.
- First-line therapy: alendronate or risedronate for medium risk; zoledronic acid or denosumab for high risk; teriparatide for very high risk (e.g., multiple or recent vertebral/hip fractures).
- In adults < 50 years, treatment should be individualized for a Z-score ≤ −2 or in the presence of fragility fractures.

**Table 1:** Risk classification of GC users based on ECTS recommendation [8].

Fracture Risk Category	Definition
Medium risk	Adults without a fracture within the past 2 years
High risk	Adults with a recent fracture (≤2 years) and/or ≥1 vertebral fracture of moderate or severe grade (Genant ≥ 2)
Very high risk	Adults aged ≥70 years with a recent hip or pelvic fracture and/or ≥1 vertebral fracture of moderate or severe grade (Genant ≥ 2)

These recommendations underline the importance of immediate risk assessment and, where indicated, the early initiation of anti-osteoporotic therapy, even without waiting for dual-energy X-ray absorptiometry (DXA) results, in order to minimize the fracture burden among GC-treated patients.

GC therapy is also explicitly recognized as a major fracture risk factor in international osteoporosis guidelines, with specific adaptations regarding diagnosis and treatment thresholds. The 2024 update of the National Osteoporosis Guideline Group (NOGG) recommends that patients aged ≥50 years starting oral GCs at doses ≥ 7.5 mg prednisolone equivalent daily for ≥3 months should receive bone-protective treatment immediately, without awaiting DXA results. In this context, FRAX^®^ probabilities are adjusted upward to reflect the dose-dependent effect of GCs on the fracture risk, and treatment initiation is advised at higher BMD levels (i.e., at “better” T-scores) compared to other populations [12].

Similarly, the 2024 consensus guideline of the Austrian Society for Bone and Mineral Research (ÖGKM) emphasizes that oral GC therapy increases the fracture risk in a dose-dependent manner, and that this risk is only partly mediated by BMD. Therefore, the ÖGKM guideline also supports arithmetic adjustments of FRAX^®^ probabilities for GC use [13].

The DVO (Dachverband Osteologie e.V.) is the German umbrella organization coordinating interdisciplinary, evidence-based guidelines for the diagnosis and treatment of osteoporosis. In their updated 2023 DVO guideline for osteoporosis management in postmenopausal women and men over 50, a more immediate 3-year risk assessment framework has been established [14]. For patients on prolonged GC therapy, the guideline strongly recommends initiating osteoanabolic treatment with teriparatide, rather than beginning with antiresorptive agents such as bisphosphonates or denosumab. This recommendation underscores the particular severity of GC-associated bone loss. Together, these recommendations highlight that GCs are one of the few secondary causes of osteoporosis with dedicated guideline adaptations, underscoring the necessity of proactive and earlier therapeutic intervention.

## 3. Aromatase Inhibitors

AIs are currently the preferred agents for adjuvant treatment in women with estrogen receptor-positive breast cancer, which accounts for approximately 80% of all breast cancers. Adjuvant endocrine therapy following tumor resection, generally administered up to 10 years, has been shown to prevent recurrence and improve survival [15]. This fact, in turn, has resulted in an increase in adverse long-term-treatment effects, including bone loss and finally osteoporotic fractures [16]. AIs reduce the body’s own estrogen production by up to 80–90% by blocking the peripheral conversion of androgens to estrogen in peripheral tissues, including fat, skin, and bone, by the enzyme aromatase [17]. AIs block this enzyme, resulting in a reduction in estrogen levels and significantly impacting bone remodeling processes. On the one hand reduced estrogen levels can help inhibit the growth of estrogen-sensitive cancer cells; on the other hand, AIs also have negative effects on bones, which are important to understanding the management of potential side effects during treatment. Physiologically, estrogen helps to balance the remodeling process by promoting osteoblast activity (bone formation) and inhibiting osteoclast activity (bone resorption). When estrogen levels drop due to aromatase inhibition, this balance is disturbed: Estrogen normally inhibits osteoclasts, the cells responsible for breaking down bone. When estrogen levels are reduced, osteoclast activity increases, leading to greater bone resorption. However, estrogen also stimulates osteoblasts (bone-forming cells). With estrogen deficiency, osteoblast activity diminishes, leading to reduced bone formation. This imbalance between resorption and formation results in a net loss of bone mass, a condition known as osteopenia (low bone mass), or, in more severe cases, osteoporosis. AIs may also affect bone through mechanisms unrelated to estrogen deficiency. For example, the drugs may influence cytokines and other signaling molecules involved in bone metabolism. However, the primary concern remains the estrogen deprivation caused by aromatase inhibition, which is the most significant factor contributing to bone loss [17].

Osteoporosis that can develop under these therapies is also known as Breast Cancer Treatment-Induced Osteoporosis (BTIOP) or AI-Associated Bone Loss (AIBL). In recent years, these substances have replaced the selective estrogen receptor modulator tamoxifen as the preferred treatment option for postmenopausal women [18,19]. The most commonly used AIs in clinical practice are exemestane, anastrozole, and letrozole: the former is steroidal, while the latter two are non-steroidal [20]. While tamoxifen is osteoprotective in postmenopausal women (the opposite effect on bone metabolism is seen in premenopausal women), AIs have negative effects on BMD and possibly also on fracture risk [15]. The interpretation and comparability of studies on the effects of AIs on the skeletal system are made very difficult by the use of tamoxifen as a comparator, partly because of study designs in which patients had tamoxifen as a prior therapy [21]. Methodologically comparable data on the effects of different AIs on BMD and fracture rates are currently lacking. Increased bone substance loss of up to 4% was shown, for example, in the ATAC study (Arimidex, tamoxifen, alone or in combination) over 2 years [22]. Similar findings were also seen in a study with letrozole versus a placebo after 5 years of tamoxifen therapy [23]. Significant increases in fracture rates were shown in several studies on postmenopausal patients with breast cancer, although the comparability of the data is again methodologically difficult due to the use of tamoxifen as a comparator and also prior therapy [24,25]. A population-based study of more than 5000 younger breast cancer patients (aged 20–39 years) in an Asian cohort (Taiwan) evaluated the fracture risk following various treatments with either AIs, radiotherapy, or monoclonal antibodies [26]. A multivariate proportional hazard analysis showed that all these treatment regimens were significantly associated with a high risk of fracture with patients who received AIs for more than 180 days at particularly high risk with a hazard ratio (HR) for fracture of 1.77. In addition, a meta-analysis of 30 randomized controlled trials (RCTs) including 117,974 breast cancer patients found a significantly higher incidence of vertebral fractures in AI users versus those not treated with AIs [27]. In this context, the relative risk (RR) for hip fractures and non-vertebral fractures was 1.18 (95%CI: 1.02–1.35; *p* < 0.001 in each case) versus controls, while the RR for vertebral fractures was 1.84 (95%CI: 1.36–2.49; *p* < 0.001). Basically, the increasing use of the extended duration of AI treatment (up to 10 years) adds to this increased fracture risk. In this context, a systematic review and meta-analysis of seven trials of 16,349 breast cancer patients treated with either extended duration AIs, placebos, or no treatment found that longer treatment with AIs was associated with a significantly higher risk of fractures (odds ratio [OR]: 1.34; 95%CI: 1.16–1.55, *p* < 0.001) [28]. The finding is confirmed by another meta-analysis of eight RCTs including 15,966 patients that found that a longer duration of AI therapy for postmenopausal patients with early breast cancer was also associated with an increased RR of low-energy fractures (RR = 1.59; RD = 0.02, *p* = 0.002) and osteoporosis (RR = 1.53; RD = 0.07, *p* = 0.005) [29].

In summary, therapy with AIs is associated with a significantly higher fracture risk than that with tamoxifen [25]. In osteoporosis management, BMD measurements are currently recommended at one- to two-year intervals for all postmenopausal women undergoing treatment with AIs [21,30].

Although the development of the FRAX^®^ tool was a significant step in osteoporotic risk assessment, it was developed for use in the general population and was not specifically designed for use in patients with breast cancer undergoing AI treatment. When using the FRAX^®^ tool, AI exposure is not a parameter that can be inputted directly but has been proposed to categorize it as “secondary osteoporosis” to reflect the associated increased risk of fracture. As was shown in a Canadian population-based study registry that included women initiating AIs for breast cancer [31], underestimation could be avoided by adding BMD values. Similar results were also obtained in a study conducted in Denmark [32].

In recent years, new measurement techniques have emerged to allow for more detailed insights into bone health and microarchitecture, like the trabecular bone score (TBS) [32] or high-resolution peripheral quantitative computed tomography (HRpQCT) [33], a three-dimensional imaging technique that enables discrimination of trabecular and cortical bone compartments, providing densitometric and structural information. Moreover, emerging biomarkers such as microRNAs (miRNAs) may provide additional insights into bone metabolism and could help predict fracture risk and monitor treatment responses [34,35]. However, further studies are needed to confirm any incremental benefits of these techniques.

From the therapeutic aspect, bisphosphonates (zoledronic acid, risedronate (35 mg)) and denosumab (60 mg) [36,37,38,39,40] can be used to prevent bone substance loss during AI medication; in addition, a significant reduction in fracture risk has been shown for almost all compounds [21].

## 4. Androgen Deprivation Therapy

ADT for prostate cancer includes a number of options, including purely surgical, bilateral orchidectomy [41] and pharmacotherapies with gonadotropin-releasing hormone (GnRH) analogs as well as purely antiandrogenic agents (cyproterone acetate, flutamide, and bicalutamide) [42]. The use of these therapies has increased considerably in recent years, and the development of osteoporosis has consequently become a frequent complication. Basically, testosterone has an anabolic effect, promoting osteoblast activity and stimulating bone formation [43,44], while having indirect effects on osteoclast activity and in reducing excessive bone resorption. As a result, testosterone influences BMD by enhancing the incorporation of minerals like calcium into bone tissue, contributing to stronger, denser bones [42,45]. Contrary to this, ADT is associated with significantly increased bone loss, leading to increased fracture rates due to the ability to disrupt the delicate balance between bone formation and resorption, leading to negative effects on bone metabolism due to the lack of testosterone’s inhibitory effect on osteoclastogenesis [43,46].

Data analyses of more than 50,000 men with ADT after prostate cancer and a minimum survival period of 5 years after diagnosis show fractures in more than 19% of patients [45]. In another retrospective study, the clinical fracture rate in men treated with GnRH analogs was calculated at 7.91/100 person-years compared to 6.55/100 person-years in men with early prostate cancer who were not treated with GnRH analogs, which corresponds to an RR of 1.23 (95%CI: 1.09–1.34). The hip fracture risk is particularly significant. However, the fracture risk is also increased in men with prostate cancer without ADT [47]. Antiresorptive osteoporosis therapies such as raloxifene, toremifene, risedronate, pamidronate, zoledronic acid, alendronate, and denosumab show similar increases in bone density and reductions in fracture risk [48,49,50,51,52].

## 5. Thyroxine

Thyroxine is used to replace hypothyroidism and suppress thyroid-stimulating hormone (TSH) in euthyroid goiter or after surgery and radioiodine therapy of differentiated thyroid carcinoma (DTC). In the follow-up treatment of DTC, the aim is to inhibit endogenous TSH production and reduce the peripheral TSH level (normal range: 0.4–4.0 mIU/L) to <0.1 mIU/L (high-risk patients) or 0.1–0.5 mIU/L (intermediate-risk patients) according to the 2016 American Thyroid Association (ATA) guidelines [53]. During TSH suppression therapy, the values for free triiodothyronine (fT3) and free thyroxine (fT4) should be within the normal range. A suppressed TSH level and normal values for fT3 and fT4 are by definition subclinical hyperthyroidism, which has an effect on bone metabolism, BMD, and fracture risk [54]. In the case of TSH suppression therapy, exogenous subclinical hyperthyroidism is present. In hyperthyroidism, T3 and T4 increase the activity of osteoblasts and osteoclasts (increased remodeling), with bone resorption predominating over bone formation. Mild hypercalcemia suppresses parathyroid hormone (PTH) and lowers 1,25(OH)vitamin D, leading to decreased intestinal calcium absorption and increased renal calcium excretion [55]. TSH also has a resorption-inhibiting effect via a TSH receptor on the osteoclasts. Suppression of TSH therefore causes an increase in bone resorption [56].

According to a meta-analysis of 17 studies, TSH suppression therapy in DTC resulted in a significant decrease in the lumbar spine BMD and a similar trend in the total hip BMD in postmenopausal women compared to controls [57]. Premenopausal women even had increased lumbar spine and total hip BMDs with TSH suppression therapy. In men, no association was found between TSH suppression therapy and BMD. Subclinical hyperthyroidism is associated with an increased risk of fractures in women and men (adjusted HR: 1.34; 95%CI: 1.09–1.65) [58]. A meta-analysis that included 70,298 individuals (men and women) in 13 cohort studies, of whom 2219 had subclinical hyperthyroidism, also found an increased fracture rate in subclinical hyperthyroidism compared to euthyroidism [59]. The HRs were 1.28 (95%CI: 1.06–1.53) for all fractures, 1.36 for hip fractures (95%CI: 1.13–1.64), and 1.51 (95%CI: 0.93–2.45) for vertebral fractures. As TSH decreased, the HR for fractures increased. Another meta-analysis, which included 313,557 people, also found an increased risk of fractures in men and women with subclinical hyperthyroidism [60]. The RR was 1.17 (95%CI: 1.08–1,26) for any fracture, 1.27 (95%CI: 1.09–1.48) for hip fracture, and 1.97 (95%CI: 1.31–2.97) for vertebral fracture. In this meta-analysis, subclinical hyperthyroidism was associated with a lower distal forearm BMD in women and a lower ultradistal forearm BMD in men and women.

## 6. Proton Pump Inhibitors (PPIs)

Long-term use of PPIs is associated with an increase in osteoporotic fracture risk [61]. In 2010, the US Food and Drug Administration (FDA) issued a warning about an increased risk of fractures (hip, wrist, spine) with PPI therapy [62]. The causes of the increased fracture rate in PPI therapy are probably multifactorial and currently not clearly understood [63,64]. A decrease in BMD, measured using the DXA method, does not appear to be the main reason for the increased fracture frequency; however, it has a negative effect on the trabecular bone (a decrease in the TBS was observed during ongoing PPI use) [65]. Another possible cause is reduced calcium absorption due to acid deficiency, with hypocalcemia then leading to secondary hyperparathyroidism with increased bone resorption. Reduced magnesium absorption can also lead to osteoclast activation and osteoblast inhibition. Taking PPIs reduces the absorption of vitamin B12, which contributes to an increased tendency to fall via neurological and muscular disorders. A vitamin B12 deficiency can lead to hyperhomocysteinemia with subsequent collagen synthesis disorders. The increase in pH during PPI therapy causes hypergastrinemia, which also leads to activation of osteoclasts via an increase in histamine production in the enterochromaffin cells. PPIs can have a direct negative effect on collagen formation in the extracellular matrix [66]. Finally, inhibition of bone-specific phosphatase (PHOSPHO1) by PPIs can impair matrix mineralization [67].

Taking PPIs for more than 1 year increases the risk of hip fractures by 20–62% and the risk of vertebral fractures by 40–60% [68]. An evaluation of several reviews revealed the following fracture probabilities with PPI therapy: an OR of 1.16–1.50 (95%CI: 1.02–1.72) for spine fractures, an OR of 1.23–1.30 (95%CI: 1.11–1.43) for hip fractures, and an OR of 1.20–1.56 (95%CI: 1.11–1.85) for any bone fracture [69]. Once the PPI is stopped, the bone damage is reversible, and the risk of fractures decreases again. Short-term use of PPIs has no negative effect on the bones.

A combination of oral bisphosphonates with PPIs reduces the fracture-inhibiting effect of bisphosphonates, thereby increasing the risk of fractures again [70]. Therefore, intravenous bisphosphonate administration should be considered in osteoporosis patients who require PPI therapy. Even with effective teriparatide therapy, the risk of new and worsened vertebral fractures is higher when PPIs are administered simultaneously, compared to teriparatide treatment in non-PPI users [71].

In summary, long-term PPI therapy is associated with a modest but significantly increased risk of fragility fractures. The risk of falls is increased with long-term PPI therapy [64], so this aspect of fracture risk must also be taken into account in clinical practice. PPI therapy should therefore only be administered if there is a clear indication and for the shortest possible duration. Particularly strict criteria must be observed when administering PPIs to patients at risk of osteoporosis.

## 7. Anticoagulants (Heparin, Vitamin K Antagonists—Coumarins, and Direct Oral Anticoagulants)

Heparin, unfractionated heparin (UFH), or low-molecular-weight heparin (LMWH) and VKAs have a relevant influence on bone metabolism [72]. No serious negative effects on bone have been demonstrated for direct oral anticoagulants (DOACs) so far.

### 7.1. Heparin

UFH and LMWH inhibit the differentiation and function of osteoblasts and thus lead to reduced bone formation. In addition, UFH causes increased bone resorption by inhibiting osteoprotegerin (OPG) activity, which results in a RANKL-induced increase in osteoclasts. No increased bone resorption has been found with LMWH in the therapeutic range, so the use of LMWH causes less damage to the bone than UFH. The histology of “heparin osteoporosis” shows rarefaction of the spongiosa with unchanged cortical bone. The resorption surfaces and number of osteoclasts are increased (UFH), and the osteoblasts are reduced (UFH and LMWH) [72,73]. High-dose long-term therapy with UFH (≥10,000 IU/day) led to a significant decrease in BMD of up to 10% in up to one third of patients, and vertebral fractures occurred in up to 15% of patients [73]. Long-term therapy with LMWH also reduces bone formation but does not lead to a significant increase in bone resorption. This results in a lower risk of osteoporosis and fewer fragility fractures than with UFH. A comparative study showed fractures in 15% of patients under UFH but only in 2.5% under LMWH after treatment over 3–6 months [74]. A meta-analysis showed that treatment with LMWH for 3–6 months did not increase the risk of fractures, but longer therapy for 3–24 months led to a decrease in bone mineral density [75]. In summary, long-term therapy with UFH reduces BMD and causes an increased fracture risk, while LMWH reduces BMD in the longer term but an increased fracture risk with LMWH has not been clearly demonstrated.

### 7.2. Vitamin K Antagonists—Coumarins

The VKAs coumarins warfarin, acenocoumarol, and phenprocoumon are used for oral anticoagulation. Coumarins are competitive inhibitors of vitamin K, which is necessary as a cofactor in the gamma-carboxylation of coagulation factors in the liver and of intact osteocalcin in bone. In the case of vitamin K deficiency or antagonism by coumarins, uncarboxylated or undercarboxylated osteocalcin is formed in the osteoblasts, which is not properly incorporated into the bone matrix. It also has a reduced calcium-binding property, resulting in impaired matrix mineralization [72]. Excessive dietary restriction of vitamin K intake during VKA therapy may lead to further bone deterioration and should therefore be avoided.

The effect of VKAs on bone density and bone quality shows controversial results in studies. A decrease in bone density is mostly observed [76], but not in all studies. Due to the well-documented negative effects of VKAs on bone metabolism, a clinically relevant decrease in BMD seems likely with long-term therapy (>1 year), especially in people with risk factors for osteoporosis. The incidence of fractures during long-term VKA therapy is increased in some studies; however, the results are not consistent. A Mayo Clinic study of women aged ≥35 years showed an increased risk of vertebral and rib fractures with VKA therapy for 1 year or longer compared to the expected fracture rates in the general population [77]. In a retrospective study of 4461 patients with long-term warfarin therapy, an increased fracture rate was found in men but not in women [78]. A meta-analysis of 23 studies on VKA therapy, which included 1,121,582 patients, found a slightly increased fracture rate in women and elderly people ≥ 65 years [79]. Long-term VKA therapy can therefore lead to an increased fracture risk, but the association is not strong.

### 7.3. Direct Oral Anticoagulants

DOACs are selective inhibitors of factor Xa (apixaban, edoxaban, rivaroxaban) or factor IIa (dabigatran) in blood coagulation. Serious negative effects of these drugs on bone metabolism are not yet known. In patients whose anticoagulation was switched from warfarin to rivaroxaban, an increase in bone alkaline phosphatase and a decrease in undercarboxylated osteocalcin were observed, indicating an improvement in bone metabolism [80]. Changes in the BMD and TBS in patients receiving DOACs (DOAC group (DOACG)) or warfarin (warfarin group (WG)) for more than 1 year were compared with a control group (CG) [81]. Low BMD was diagnosed in 42% of the CG, 50% of the DOACG, and 66% of the WG. Thus, there were slightly more patients with low BMD in the DOACG than in the CG, but the proportion of patients with low BMD was significantly higher in the WG. The TBS decreased in the same order: CG > DOACG > WG. An increase in the risk of fractures with DOAC therapy has not been demonstrated. In several studies, the frequency of fractures when administering VKAs is compared with the administration of DOACs. A meta-analysis including 455,343 patients with atrial fibrillation or venous thromboembolism found that the risk of any fracture compared with VKAs was the lowest with apixaban (RR: 0.59; 95%CI: 0.48–0.73), followed by rivaroxaban (RR: 0.72; 95%CI: 0.60–0.86), edoxaban (RR: 0.88; 95%CI: 0.62–1.23), and dabigatran (RR: 0.90; 95%CI: 0.75–1.07) [82].

Conclusion: In clinical practice, DOACs are generally safer with regard to bone health compared to heparin or VKAs and should be preferred from an osteological point of view if there is no contraindication (e.g., pregnancy). When selecting the drug for long-term anticoagulation with LMWH, VKAs, or DOACs, consideration should be given to whether the patient is at an increased risk of fracture.

## 8. Antidepressants—Non-Selective Serotonin and/or Noradrenaline Reuptake Inhibitors, Selective Serotonin Reuptake Inhibitors

Antidepressants increase the neurotransmitters serotonin and noradrenaline in the synaptic cleft, since depression is caused by a deficiency in these neurotransmitters in certain synapses. This occurs either via an inhibition of the presynaptic reuptake of serotonin or noradrenaline by reuptake inhibitors by increasing the release of noradrenaline through α_2_-antagonists, or by inhibiting the degradation of serotonin and noradrenaline by monoamine oxidase (MAO) inhibitors. A distinction is made between non-selective serotonin and/or noradrenaline reuptake inhibitors (NSRIs), which inhibit the reuptake of serotonin and/or noradrenaline and are antagonists at other neurotransmitter receptors (tricyclic antidepressants (TCAs), e.g., amitriptyline), and selective serotonin reuptake inhibitors (SSRIs), which selectively inhibit the reuptake of serotonin (e.g., citalopram, fluoxetine). Antidepressants are a risk factor for osteoporosis [83].

Osteoclasts and osteoblasts express serotonin receptors and serotonin transporters. SSRIs are detected in high concentrations in the bone marrow. Most SSRIs inhibit osteoclast formation and resorption by osteoclasts in a dose-dependent manner [84]. SSRIs (except citalopram) inhibit the formation of alkaline phosphatase and mineralization by osteoblasts. SSRIs induce apoptosis of osteoclasts and osteoblasts. Ultimately, a “low bone turnover” condition occurs, which can lead to BMD loss and an increased risk of fragility fractures. Antidepressants also influence the differentiation of human mesenchymal stem cells (hMSCs) to osteoblasts and adipocytes [85]. An inhibitory effect of fluoxetine on the mineralization during the differentiation of hMSCs into osteoblasts has been shown.

Antidepressants can affect bone metabolism and BMD. The bone remodeling markers C-telopeptide (CTX–resorption) and procollagen type 1 N-terminal propeptide (P1NP–formation) were investigated in men under SSRI therapy [86]. In younger men (20–60 years) under SSRI therapy, CTX (by 12%) and P1NP (by 13.6%) were significantly lower than in non-users. No difference in the bone remodeling markers was seen in older men (61–94 years) with and without SSRI therapy. In postmenopausal women (57–67 years), an accelerated decrease in the BMD in the femoral neck was observed during treatment with SSRIs and TCAs [87]. A meta-analysis of 42,656 patients with depression found a significant decrease in BMD under SSRI therapy [88]. A reduction in the TBS and thus a deterioration in bone texture was observed in women receiving SSRIs or TCAs [89].

An increased risk of major osteoporotic fractures (adjusted HR: 1.43; 95%CI: 1.27–1.60) and hip fractures (aHR: 1.48; 95%CI: 1.18–1.85) was found in men and women (≥40 years) taking SSRIs [90]. A meta-analysis of 34 studies found an increased risk for all fracture types (RR: 1.39; 95%CI: 1.32–1.47) among antidepressant users (SSRIs and TCAs) compared to non-users, with SSRIs leading to a higher increase in fractures than TCAs [91]. Another meta-analysis of 38 studies found a significant association for antidepressant therapy with fragility fractures of the hip (OR: 2.07; 95%CI: 1.98–2.17) [92]. The increase in the fracture risk is the greatest at the start of antidepressant therapy, with a maximum within 1 month for TCAs and after 8 months for SSRIs, and the increased risk of fracture due to antidepressants is reduced to the baseline value in the year after discontinuation of medication [83].

Even after adjustment for confounders, there is a link between the use of antidepressants and increased fracture risk [83]. In clinical practice, monitoring of bone health is recommended in patients receiving SSRIs [83,88].

## 9. Neuroleptics (Antipsychotics)

The antipsychotic effect of neuroleptics is primarily based on an antagonism to the dopamine receptor, meaning that they antagonize the dopaminergic hyperfunction in schizophrenia. Classical neuroleptics (“first generation”) act as dopamine D_2_ receptor antagonists (phenothiazines, thioxanthenes, butyrophenones). Atypical neuroleptics (“second generation”) act less strongly via D_2_ receptors and more via D_4_ and/or 5-HT_2A_ receptors (e.g., clozapine, olanzapine, risperidone).

As dopamine receptor antagonists, neuroleptics reverse the dopamine-related inhibition of prolactin secretion in the pituitary gland and thus lead to hyperprolactinemia [93]. Via prolactin receptors on osteoblasts, prolactin releases dose-dependent cytokines, such as RANKL or tumor necrosis factor (TNF)-α, and the osteoprotegerin mRNA level is downregulated. An increase in osteoclast activity is therefore the result of hyperprolactinemia. An increased prolactin level also inhibits the proliferation of osteoblasts. The hypothalamic secretion pulsatility of gonadotropin-releasing hormone is disturbed by hyperprolactinemia. This leads to the inhibition of secretion of the gonadotropins luteinizing hormone (LH) and follicle-stimulating hormone (FSH) in the pituitary gland and thus to secondary hypogonadism, which is also a risk for osteoporosis [93]. A very high increase in prolactin is induced with, e.g., haloperidol, amisulpride, and risperidone, whereas quetiapine or clozapine cause a smaller increase in prolactin [94].

In an osteoblast cell culture, atypical neuroleptics (olanzapine, risperidone, amisulpride, or aripiprazole) caused a decrease in proliferation and an increase in apoptosis of osteoblasts [95]. A decrease in β-catenin expression in the cells indicated the involvement of Wnt/β-catenin signaling in this process, which can contribute to the development of osteoporosis under atypical neuroleptics.

The increase in the bone resorption marker serum β-crosslaps indicates increased bone resorption during treatment with atypical neuroleptics in patients with schizophrenia compared to untreated patients [96]. There was no difference in the formation marker serum osteocalcin between treated and untreated patients. An 11% lower BMD in the lumbar spine and a 9.9% lower BMD in the hip were found in women under 60 years of age who received neuroleptics compared to women under 60 years of age who did not receive neuroleptics [97]. In women aged 60 years and over and in men, there was no difference in the BMD with and without neuroleptic therapy. Antipsychotic medication is also associated with a deterioration in parameters of the quantitative heel ultrasound (“broadband ultrasound attenuation” (BUA)) and “stiffness index” (SI) [98].

Studies on fracture risk predominantly show an increased risk of fractures with neuroleptic therapy. For example, a study from Taiwan showed an increased risk of hip fractures with neuroleptic therapy in patients with schizophrenia (adjusted OR: 1.61; 95%CI: 1.24–2.10) [99]. At the treatment start, the fracture risk was significantly higher than that later on. The fracture risk was significantly increased with classical (first-generation) neuroleptics but not with atypical (second-generation) neuroleptics. A review with a meta-analysis of 19 studies (544,811 people) showed the following ORs for the occurrence of hip fractures during neuroleptic therapy compared to controls: 1.67 (95%CI: 1.45–1.93) for typical (first-generation) neuroleptics, 1.33 (95%CI: 1.11–1.58) for atypical (second-generation) neuroleptics, and 1.46 (95%CI: 1.31–1.64) for typical and atypical neuroleptics combined [100]. Two other meta-analyses also showed an increased risk of hip fractures with neuroleptic therapy [92,101].

Patients receiving neuroleptics have drug-induced hyperprolactinemia, which has a multifactorial negative impact on bone [93]. Therefore, bone health should be monitored during antipsychotic therapy.

Lithium salts are mood stabilizers and are used in the treatment of bipolar disorders. Lithium chloride stimulates osteogenesis by human bone marrow-derived mesenchymal stem cells while simultaneously inhibiting adipogenesis by acting on the Wnt-signaling and Hedgehog-signaling pathways [102]. Lithium also suppresses the activity of osteoclasts by inhibiting the RANKL/OPG system [103]. Therefore, lithium preparations are considered bone-protecting substances, in contrast to neuroleptics. Patients with bipolar disorder treated with lithium preparations showed a reduced risk of osteoporosis compared to those without lithium therapy [104]. A meta-analysis showed a 20% reduction in the fracture risk (RR: 0.80; 95%CI: 0.73–0.87) with lithium therapy compared to control subjects [105].

## 10. Antidiabetic Drugs—Thiazolidinediones

TZDs, including pioglitazone and rosiglitazone, increase insulin sensitivity through activation of the peroxisome proliferator-activated receptor γ (PPARγ). This activation exerts catabolic effects on bone at multiple levels. In bone marrow stromal cells (BMSCs), adipogenesis is stimulated while osteoblastic differentiation is inhibited [106,107]. Consequently, the differentiation balance of osteoblastic precursor cells shifts toward adipocytes. At the same time, rosiglitazone suppresses the expression of osteoblast-specific genes, resulting in reduced bone formation, enhanced osteoclastogenesis, and increased osteocyte apoptosis [108,109]. These pathophysiological mechanisms are reflected in clinical data. In the large ADOPT trial (A Diabetes Outcome Progression Trial), rosiglitazone was associated with approximately double the fracture incidence compared with metformin, particularly in pre- and postmenopausal women [109]. A population-based cohort study found that exposure to rosiglitazone or pioglitazone was associated with a significantly increased risk of hospitalization for hip fracture (ORs: 1.16 and 1.18, respectively) [110]. A meta-analysis confirmed these findings, demonstrating an approximately twofold increase in the fracture risk, independent of age, sex, or treatment duration [111]. Notably, the loss of BMD under TZDs was not reversible after discontinuation of therapy [112]. Clinical studies have also documented decreases in bone formation markers (e.g., osteocalcin, PINP) and increases in bone resorption markers (e.g., CTX), underscoring the shift toward a catabolic bone turnover state [108]. In summary, TZDs promote adipogenic signaling and inhibit osteoblastic processes via PPARγ activation, leading to reduced bone formation, increased bone resorption, and elevated fracture risk. In postmenopausal women with type 2 diabetes, TZD therapy should therefore be critically evaluated and bone metabolism monitored regularly. The development of new TZD derivatives with improved safety profiles remains an important research objective to preserve the metabolic benefits of this drug class without compromising bone health.

Among antidiabetic agents, metformin shows the strongest evidence for beneficial skeletal effects. Experimental models demonstrate the AMPK- and BMP-2/RUNX2-mediated stimulation of osteoblastogenesis, improved OPG/RANKL signaling, and cytoprotective effects on mesenchymal stem cells [113]. Clinically, metformin use is associated with a reduced fracture risk, higher BMD, and lower osteoporosis incidence across multiple cohorts [114,115].

Dipeptidyl Peptidase-4 (DPP-4) inhibitors appear bone-neutral to mildly protective. Elevated DPP-4 activity correlates with low BMD and fractures [116], while preclinical studies show improved bone architecture and OPG/RANKL balance [117]. Clinical trials generally report neutral effects, with a possible trend toward reduced fractures [118].

Glucagon-like Peptide-1 (GLP-1) receptor agonists are overall bone-safe. Mechanistic studies show enhanced osteoblast activity and Wnt/β-catenin signaling [119]. Recent evidence suggests modest improvements in BMD and turnover markers without increased fracture risk, and a lower incidence of osteoporosis in real-world data [120,121].

Sodium-glucose Cotransporter-2 (SGLT-2) inhibitors do not increase fracture risk, despite early concerns about phosphate–FGF-23–PTH signaling [122]. Large outcome trials and meta-analyses consistently show neutral skeletal effects, with some real-world analyses suggesting no higher fracture risk compared with DPP-4 inhibitors or sulfonylureas [123,124,125,126].

In contrast, sulfonylureas and insulin are pharmacologically bone-neutral but associated with higher fracture rates due to hypoglycemia-related falls, especially in older individuals [127,128]. Insulin has anabolic actions on bone [129], and the increased fracture risk under therapy is considered indirect rather than drug-specific.

Overall, metformin shows the most consistent osteoprotective profile; GLP-1 receptor agonists and SGLT-2 inhibitors appear neutral to beneficial; DPP-4 inhibitors are bone-safe; and sulfonylureas and insulin warrant caution due to the hypoglycemia-mediated fall risk.

A limitation of this review is that it provides a selective overview and does not cover all medications potentially associated with bone loss or fracture risk (e.g., calcineurin inhibitors, loop diuretics, and anti-epileptic drugs). Selected medications associated with adverse skeletal effects and estimated fracture risk are summarized in Table 2.

## 11. Conclusions

Drug-induced osteoporosis is a frequent but often underestimated contributor to secondary osteoporosis and fragility fractures. Among the wide range of potentially bone-damaging agents, systemic GCs remain the most clinically relevant medication class, characterized by a rapid and dose-dependent increase in vertebral and non-vertebral fracture risks and supported by dedicated international guideline recommendations for early risk assessment and treatment initiation. In routine practice, therapies that induce hypogonadism represent the second major group with high fracture relevance, particularly AIs in breast cancer and ADT in prostate cancer, where bone loss is substantial and fracture prevention should be integrated into oncologic care. Beyond these, thiazolidinediones and antipsychotics warrant special attention due to consistent fracture signals, either through direct effects on bone formation or through endocrine changes and fall-related mechanisms. Other widely prescribed drug classes such as PPIs, thyroxine in overtreatment/TSH suppression, and VKAs appear to confer more modest but clinically meaningful risk increases, particularly when exposure is prolonged and the baseline fracture risk is already elevated. For anticoagulation, UFH carries a clear osteoporotic risk, whereas LMWH and especially DOACs seem to be comparatively more bone-safe options in long-term treatment decisions.

Overall, the clinical challenge is not only the individual drug effect but the frequent risk clustering—older age, comorbid inflammatory disease, reduced mobility, falls, and combinations of bone-active medications. Therefore, bone health considerations should be incorporated into long-term prescribing: identifying high-risk patients early, estimating the fracture probability (e.g., FRAX^®^), evaluating the BMD and vertebral status when indicated, correcting calcium/vitamin D deficits, and initiating guideline-based osteoporosis therapy when thresholds are met. A pragmatic prioritization of drug classes with the strongest evidence and largest clinical impact may help target preventive efforts and reduce avoidable fractures in daily practice.

## Figures and Tables

**Table 2:** Selected medications associated with adverse skeletal effects and estimated fracture risk relevance. Qualitative clinical orientation based on available evidence (BMD and fracture outcomes); not a formal risk score.

Drug Class (Examples)	Key Mechanism (s)	Main Skeletal Outcome	Fracture Risk Relevance
Glucocorticoids	↓ bone formation, ↑ resorption; impaired Ca/vitamin D metabolism	Rapid BMD loss; ↑ vertebral & hip fractures	Strong
Aromatase inhibitors	Estrogen depletion → ↑ resorption	Marked BMD loss; ↑ fractures	Strong
Androgen deprivation therapy	Hypogonadism → high bone turnover	Marked BMD loss; ↑ fractures	Strong
Thiazolidinediones	PPARγ activation → ↓ osteoblastogenesis	BMD loss; ↑ fractures (esp. women)	Relevant
Antipsychotics/neuroleptics	Hyperprolactinemia; sedation/falls	BMD loss; ↑ fractures	Relevant
Heparin (UFH > LMWH)	↓ formation (UFH pronounced)	BMD loss; ↑ fractures (UFH)	Relevant (UFH)/Moderate (LMWH)
Antidepressants (SSRIs/SNRIs)	Falls; possible direct bone effects	↑ fractures (often > BMD effect)	Moderate–relevant
Thyroxine (overtreatment)	Increased turnover (TSH suppression)	BMD loss; ↑ fractures (modest)	Moderate
Vitamin K antagonists	Impaired γ-carboxylation of bone proteins	Possible bone quality effects	Moderate
Proton pump inhibitors	↓ calcium absorption; possible fall risk	Modest fracture association	Moderate

## Data Availability

No new data were created or analyzed in this study.

## Abbreviations

GCs	Glucocorticoids
BMD	Bone mineral density
AIs	Aromatase inhibitors
ADT	Androgen-deprivation therapy
PPIs	Proton pump inhibitors
VKAs	Vitamin K antagonists
TZDs	Thiazolidinediones
GIOP	Glucocorticoid-induced osteoporosis
BMP	Bone morphogenetic protein(s)
RANKL	Receptor Activator of Nuclear Factor κB Ligand
ECTS	European Calcified Tissue Society
FRAX^®^	Fracture risk assessment
DXA	Dual-energy X-ray absorptiometry
NOGG	National Osteoporosis Guideline Group
ÖGKM	Austrian Society for Bone and Mineral Research
DVO	Dachverband Osteologie e.V.
BTIOP	Breast cancer treatment-induced osteoporosis
AIBL	Aromatase inhibitor-associated bone loss
HR	Hazard ratio
RCTs	Randomized controlled trials
RR	Relative risk
OR	Odds ratio
TBS	Trabecular bone score
HRpQCT	High-resolution peripheral quantitative computed tomography
miRNAs	MicroRNAs
GnRH	Gonadotropin-releasing hormone
TSH	Thyroid-stimulating hormone
DTC	Differentiated thyroid carcinoma
ATA	American Thyroid Association
PTH	Parathyroid hormone
FDA	US Food and Drug Administration
UFH	Unfractionated heparin
LMWH	Low-molecular-weight heparin
DOACs	Direct oral anticoagulants
OPG	Osteoprotegerin
DOACG	DOAC group
WG	Warfarin group
CG	Control group
MAO	Monoamine oxidase
TCAs	Tricyclic antidepressants
NSRIs	Non-selective serotonin and/or noradrenaline reuptake inhibitors
SSRIs	Selective serotonin reuptake inhibitors
hMSCs	Human mesenchymal stem cells
CTX	C-telopeptide
PINP	Procollagen type 1 N-terminal propeptide
TNF-α	Tumor necrosis factor
LH	Luteinizing hormone
FSH	Follicle-stimulating hormone
BUA	Broadband ultrasound attenuation
PPARγ	Peroxisome proliferator-activated receptor γ
ADOPT	A Diabetes Outcome Progression Trial
DPP4	Dipeptidyl Peptidase-4
GLP-1	Glucagon-like Peptide-1
SGLT-2	Sodium-glucose Cotransporter-2
FGF-23	Fibroblast growth factor 23
